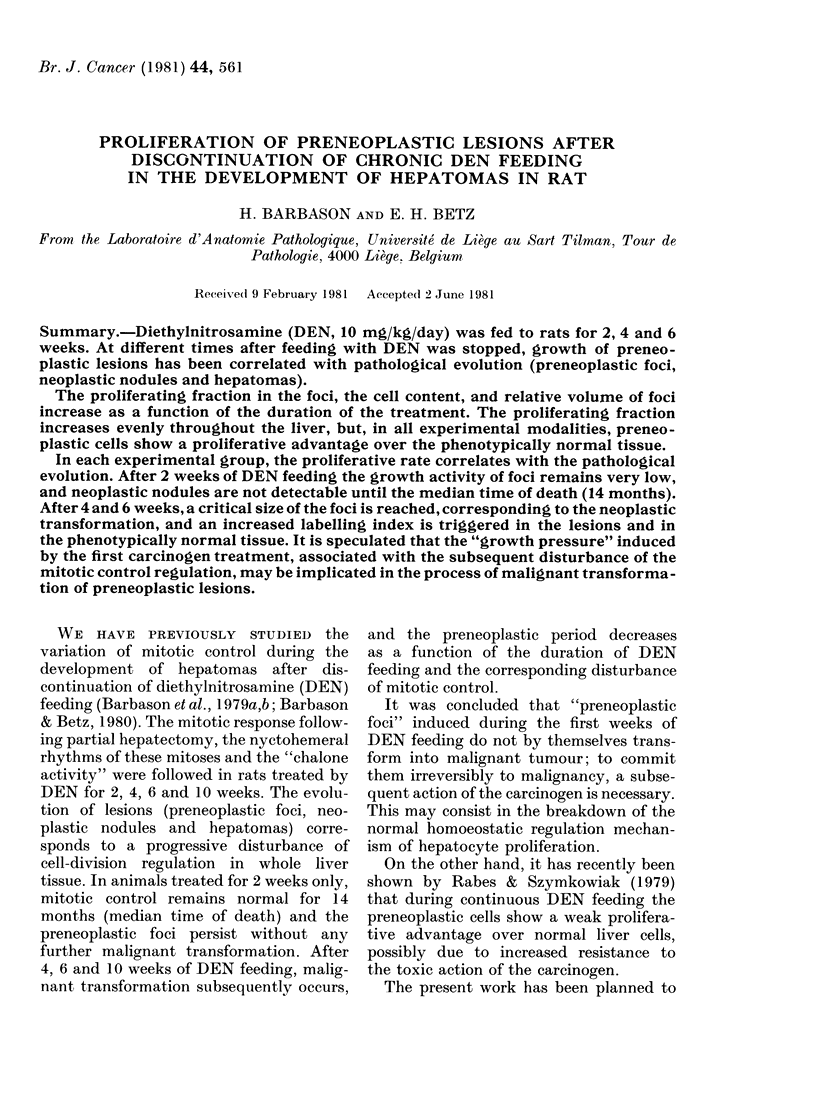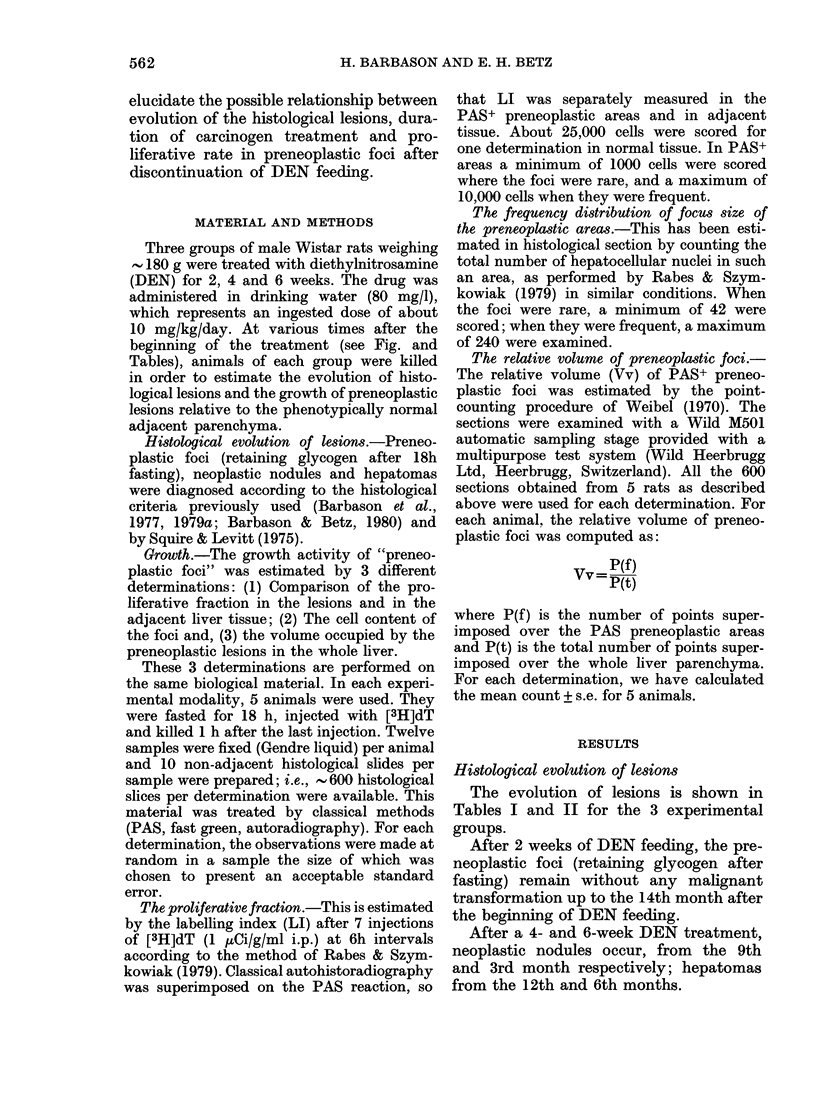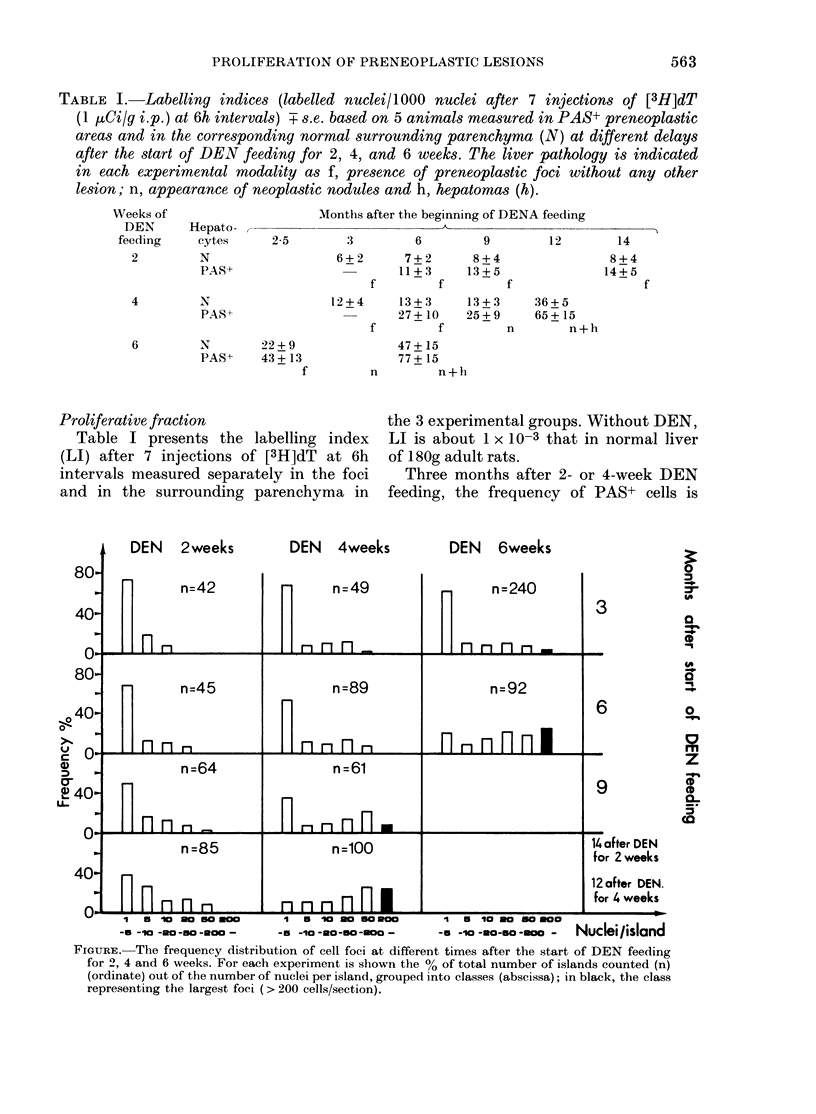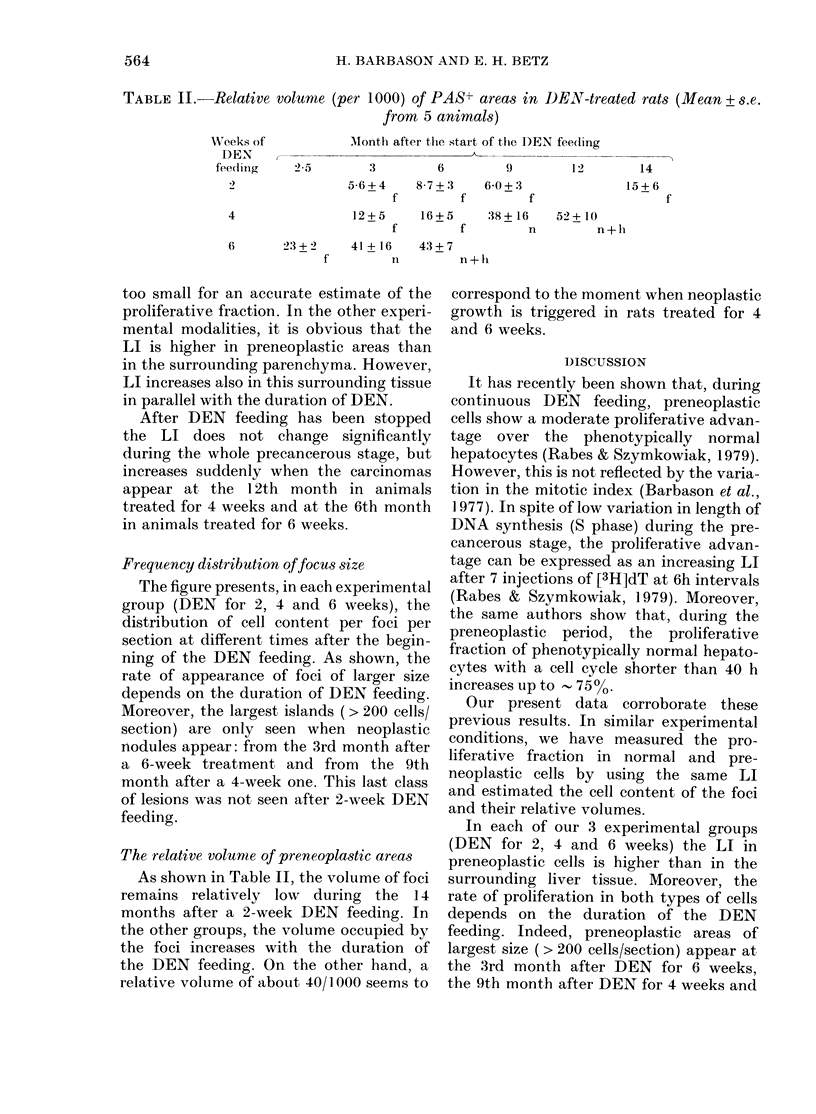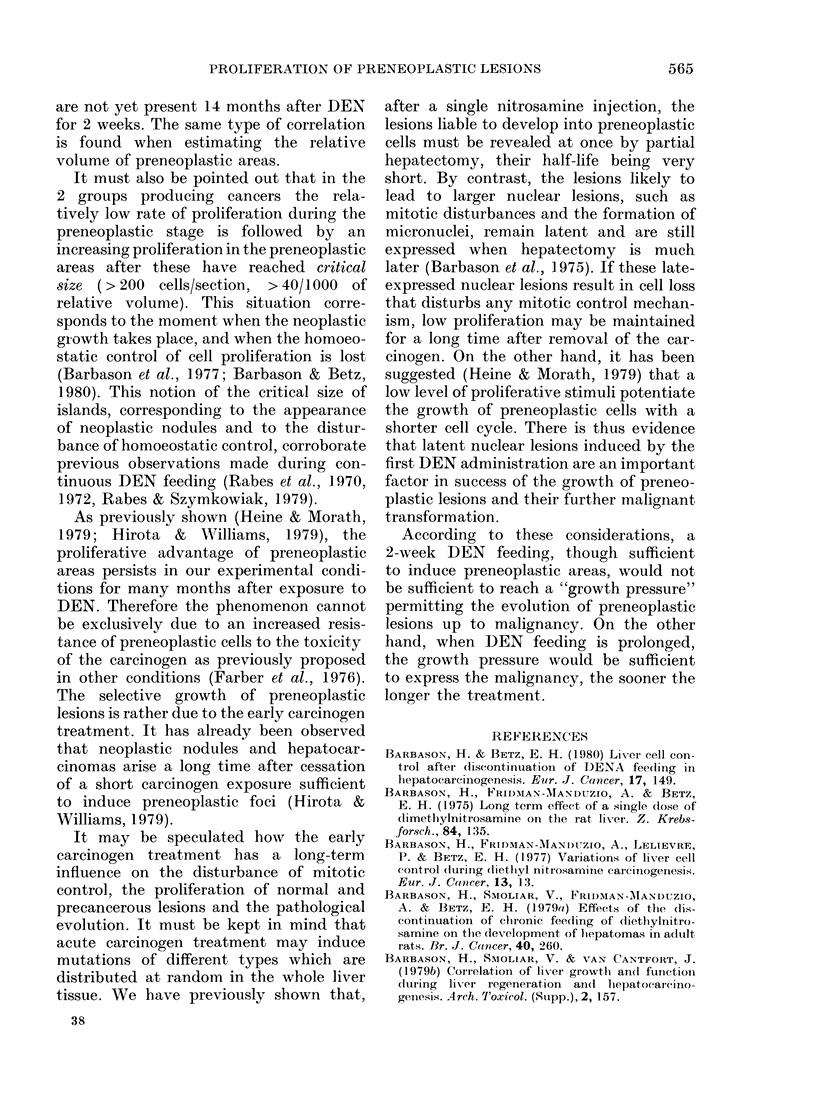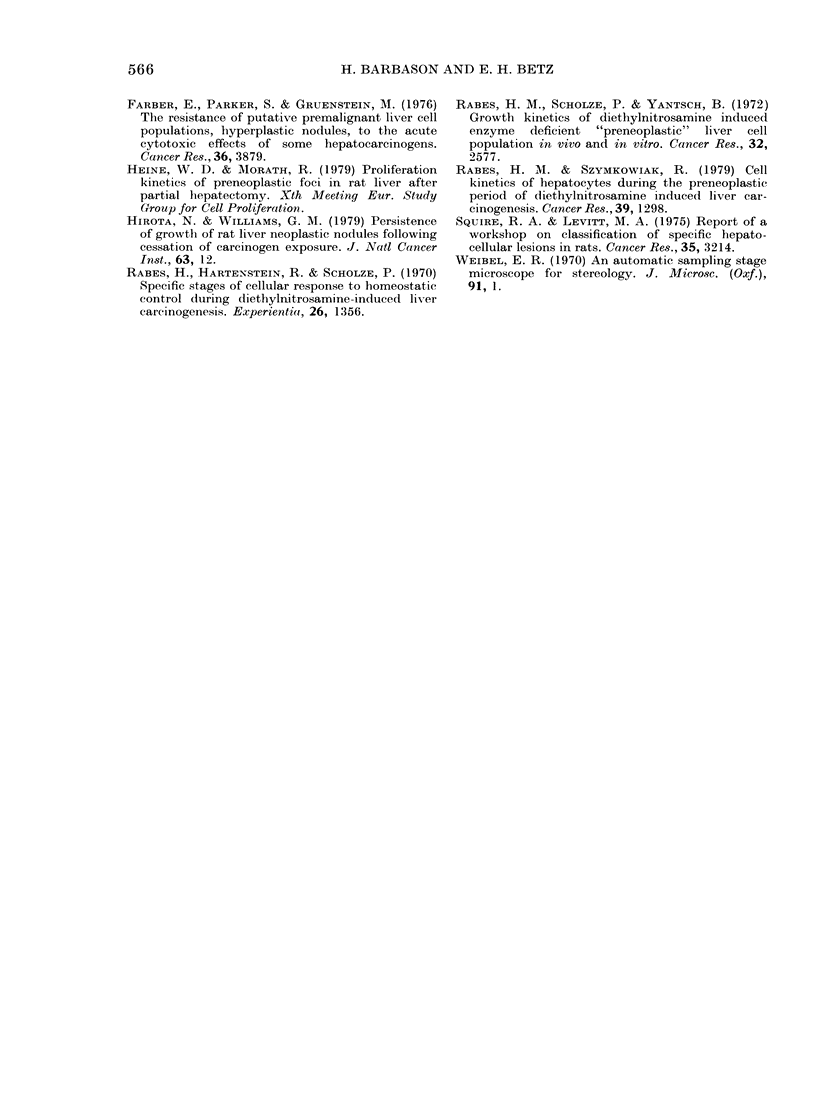# Proliferation of preneoplastic lesions after discontinuation of chronic DEN feeding in the development of hepatomas in rat.

**DOI:** 10.1038/bjc.1981.226

**Published:** 1981-10

**Authors:** H. Barbason, E. H. Betz

## Abstract

Diethylnitrosamine (DEN, 10 mg/kg/day) was fed to rats for 2, 4 and 6 weeks. At different times after feeding with DEN was stopped, growth of preneoplastic lesions has been correlated with pathological evolution (preneoplastic foci, neoplastic nodules and hepatomas). The proliferating fraction in the foci, the cell content, and relative volume of foci increase as a function of the duration of the treatment. The proliferating fraction increases evenly throughout the liver, but, in all experimental modalities, preneoplastic cells show a proliferative advantage over the phenotypically normal tissue. In each experimental group, the proliferative rate correlates with the pathological evolution. After 2 weeks of DEN feeding the growth activity of foci remains very low, and neoplastic nodules are not detectable until the median time of death (14 months). After 4 and 6 weeks, a critical size of the foci is reached, corresponding to the neoplastic transformation, and an increased labelling index is triggered in the lesions and in the phenotypically normal tissue. It is speculated that the "growth pressure" induced by the first carcinogen treatment, associated with the subsequent disturbance of the mitotic control regulation, may be implicated in the process of malignant transformation of preneoplastic lesions.


*Br. J. Cancer* (1981) **44**, 561

### PROLIFERATION OF PRENEOPLASTIC LESIONS AFTER DISCONTINUATION OF CHRONIC DEN FEEDING IN THE DEVELOPMENT OF HEPATOMAS IN RAT

H. BARBASON AND E. H. BETZ

*From the Laboratoire d'Anatomie Pathologique, Université de Liège au Sart Tilman, Tour de Pathologie, 4000 Liège, Belgium*

Received 9 February 1981 Accepted 2 June 1981

**Summary.—Diethylnitrosamine (DEN, 10 mg/kg/day) was fed to rats for 2, 4 and 6 weeks. At different times after feeding with DEN was stopped, growth of preneoplastic lesions has been correlated with pathological evolution (preneoplastic foci, neoplastic nodules and hepatomas).**

**The proliferating fraction in the foci, the cell content, and relative volume of foci increase as a function of the duration of the treatment. The proliferating fraction increases evenly throughout the liver, but, in all experimental modalities, preneoplastic cells show a proliferative advantage over the phenotypically normal tissue.**

**In each experimental group, the proliferative rate correlates with the pathological evolution. After 2 weeks of DEN feeding the growth activity of foci remains very low, and neoplastic nodules are not detectable until the median time of death (14 months). After 4 and 6 weeks, a critical size of the foci is reached, corresponding to the neoplastic transformation, and an increased labelling index is triggered in the lesions and in the phenotypically normal tissue. It is speculated that the "growth pressure" induced by the first carcinogen treatment, associated with the subsequent disturbance of the mitotic control regulation, may be implicated in the process of malignant transformation of preneoplastic lesions.**

WE HAVE PREVIOUSLY STUDIED the variation of mitotic control during the development of hepatomas after discontinuation of diethylnitrosamine (DEN) feeding (Barbason *et al.*, 1979*a,b*; Barbason & Betz, 1980). The mitotic response following partial hepatectomy, the nyctohemeral rhythms of these mitoses and the "chalone activity" were followed in rats treated by DEN for 2, 4, 6 and 10 weeks. The evolution of lesions (preneoplastic foci, neoplastic nodules and hepatomas) corresponds to a progressive disturbance of cell-division regulation in whole liver tissue. In animals treated for 2 weeks only, mitotic control remains normal for 14 months (median time of death) and the preneoplastic foci persist without any further malignant transformation. After 4, 6 and 10 weeks of DEN feeding, malignant transformation subsequently occurs, and the preneoplastic period decreases as a function of the duration of DEN feeding and the corresponding disturbance of mitotic control.

It was concluded that "preneoplastic foci" induced during the first weeks of DEN feeding do not by themselves transform into malignant tumour; to commit them irreversibly to malignancy, a subsequent action of the carcinogen is necessary. This may consist in the breakdown of the normal homoeostatic regulation mechanism of hepatocyte proliferation.

On the other hand, it has recently been shown by Rabes & Szymkowiak (1979) that during continuous DEN feeding the preneoplastic cells show a weak proliferative advantage over normal liver cells, possibly due to increased resistance to the toxic action of the carcinogen.

The present work has been planned to elucidate the possible relationship between evolution of the histological lesions, duration of carcinogen treatment and proliferative rate in preneoplastic foci after discontinuation of DEN feeding.

### MATERIAL AND METHODS

Three groups of male Wistar rats weighing ~180 g were treated with diethylnitrosamine (DEN) for 2, 4 and 6 weeks. The drug was administered in drinking water (80 mg/l), which represents an ingested dose of about 10 mg/kg/day. At various times after the beginning of the treatment (see Fig. and Tables), animals of each group were killed in order to estimate the evolution of histological lesions and the growth of preneoplastic lesions relative to the phenotypically normal adjacent parenchyma.

*Histological evolution of lesions.*—Preneoplastic foci (retaining glycogen after 18h fasting), neoplastic nodules and hepatomas were diagnosed according to the histological criteria previously used (Barbason *et al.*, 1977, 1979*a*; Barbason & Betz, 1980) and by Squire & Levitt (1975).

*Growth.*—The growth activity of "preneoplastic foci" was estimated by 3 different determinations: (1) Comparison of the proliferative fraction in the lesions and in the adjacent liver tissue; (2) The cell content of the foci and, (3) the volume occupied by the preneoplastic lesions in the whole liver.

These 3 determinations are performed on the same biological material. In each experimental modality, 5 animals were used. They were fasted for 18 h, injected with [$^3$H]dT and killed 1 h after the last injection. Twelve samples were fixed (Gendre liquid) per animal and 10 non-adjacent histological slides per sample were prepared; *i.e.*, ~600 histological slices per determination were available. This material was treated by classical methods (PAS, fast green, autoradiography). For each determination, the observations were made at random in a sample the size of which was chosen to present an acceptable standard error.

*The proliferative fraction.*—This is estimated by the labelling index (LI) after 7 injections of [$^3$H]dT (1 $\mu$Ci/g/ml i.p.) at 6h intervals according to the method of Rabes & Szymkowiak (1979). Classical autohistoradiography was superimposed on the PAS reaction, so that LI was separately measured in the PAS$^+$ preneoplastic areas and in adjacent tissue. About 25,000 cells were scored for one determination in normal tissue. In PAS$^+$ areas a minimum of 1000 cells were scored where the foci were rare, and a maximum of 10,000 cells when they were frequent.

*The frequency distribution of focus size of the preneoplastic areas.*—This has been estimated in histological section by counting the total number of hepatocellular nuclei in such an area, as performed by Rabes & Szymkowiak (1979) in similar conditions. When the foci were rare, a minimum of 42 were scored; when they were frequent, a maximum of 240 were examined.

*The relative volume of preneoplastic foci.*—The relative volume (Vv) of PAS$^+$ preneoplastic foci was estimated by the point-counting procedure of Weibel (1970). The sections were examined with a Wild M501 automatic sampling stage provided with a multipurpose test system (Wild Heerbrugg Ltd, Heerbrugg, Switzerland). All the 600 sections obtained from 5 rats as described above were used for each determination. For each animal, the relative volume of preneoplastic foci was computed as:

$$Vv = \frac{P(f)}{P(t)}$$

where P(f) is the number of points superimposed over the PAS preneoplastic areas and P(t) is the total number of points superimposed over the whole liver parenchyma. For each determination, we have calculated the mean count ± s.e. for 5 animals.

### RESULTS

### *Histological evolution of lesions*

The evolution of lesions is shown in Tables I and II for the 3 experimental groups.

After 2 weeks of DEN feeding, the preneoplastic foci (retaining glycogen after fasting) remain without any malignant transformation up to the 14th month after the beginning of DEN feeding.

After a 4- and 6-week DEN treatment, neoplastic nodules occur, from the 9th and 3rd month respectively; hepatomas from the 12th and 6th months.

TABLE I.—*Labelling indices (labelled nuclei/1000 nuclei after 7 injections of [$^3H$]dT (1 μCi/g i.p.) at 6h intervals) ∓ s.e. based on 5 animals measured in PAS$^+$ preneoplastic areas and in the corresponding normal surrounding parenchyma (N) at different delays after the start of DEN feeding for* 2, 4, *and* 6 *weeks. The liver pathology is indicated in each experimental modality as* f, *presence of preneoplastic foci without any other lesion;* n, *appearance of neoplastic nodules and* h, *hepatomas (h).*

| Weeks of DEN feeding | Hepatocytes | Months after the beginning of DENA feeding | | | | | |
|---|---|---|---|---|---|---|---|
| | | 2·5 | 3 | 6 | 9 | 12 | 14 |
| 2 | N | | 6 ± 2 | 7 ± 2 | 8 ± 4 | | 8 ± 4 |
| | PAS$^+$ | | — | 11 ± 3 | 13 ± 5 | | 14 ± 5 |
| | | | f | f | f | | f |
| 4 | N | | 12 ± 4 | 13 ± 3 | 13 ± 3 | 36 ± 5 | |
| | PAS$^+$ | | — | 27 ± 10 | 25 ± 9 | 65 ± 15 | |
| | | | f | f | n | n + h | |
| 6 | N | 22 ± 9 | | 47 ± 15 | | | |
| | PAS$^+$ | 43 ± 13 | | 77 ± 15 | | | |
| | | f | n | n + h | | | |

*Proliferative fraction*

Table I presents the labelling index (LI) after 7 injections of [$^3$H]dT at 6h intervals measured separately in the foci and in the surrounding parenchyma in the 3 experimental groups. Without DEN, LI is about $1 \times 10^{-3}$ that in normal liver of 180g adult rats.

Three months after 2- or 4-week DEN feeding, the frequency of PAS$^+$ cells is

FIGURE.—The frequency distribution of cell foci at different times after the start of DEN feeding for 2, 4 and 6 weeks. For each experiment is shown the % of total number of islands counted (n) (ordinate) out of the number of nuclei per island, grouped into classes (abscissa); in black, the class representing the largest foci ( > 200 cells/section).

TABLE II.—*Relative volume (per* 1000) *of* $PAS^{+}$ *areas in DEN-treated rats (Mean* ± *s.e. from* 5 *animals)*

| Weeks of DEN feeding | Month after the start of the DEN feeding | | | | | |
|---|---|---|---|---|---|---|
| | 2·5 | 3 | 6 | 9 | 12 | 14 |
| 2 | | 5·6 ± 4 f | 8·7 ± 3 f | 6·0 ± 3 f | | 15 ± 6 f |
| 4 | | 12 ± 5 f | 16 ± 5 f | 38 ± 16 n | 52 ± 10 n + h | |
| 6 | 23 ± 2 f | 41 ± 16 n | 43 ± 7 n + h | | | |

too small for an accurate estimate of the proliferative fraction. In the other experimental modalities, it is obvious that the LI is higher in preneoplastic areas than in the surrounding parenchyma. However, LI increases also in this surrounding tissue in parallel with the duration of DEN.

After DEN feeding has been stopped the LI does not change significantly during the whole precancerous stage, but increases suddenly when the carcinomas appear at the 12th month in animals treated for 4 weeks and at the 6th month in animals treated for 6 weeks.

*Frequency distribution of focus size*

The figure presents, in each experimental group (DEN for 2, 4 and 6 weeks), the distribution of cell content per foci per section at different times after the beginning of the DEN feeding. As shown, the rate of appearance of foci of larger size depends on the duration of DEN feeding. Moreover, the largest islands ( > 200 cells/section) are only seen when neoplastic nodules appear: from the 3rd month after a 6-week treatment and from the 9th month after a 4-week one. This last class of lesions was not seen after 2-week DEN feeding.

*The relative volume of preneoplastic areas*

As shown in Table II, the volume of foci remains relatively low during the 14 months after a 2-week DEN feeding. In the other groups, the volume occupied by the foci increases with the duration of the DEN feeding. On the other hand, a relative volume of about 40/1000 seems to correspond to the moment when neoplastic growth is triggered in rats treated for 4 and 6 weeks.

DISCUSSION

It has recently been shown that, during continuous DEN feeding, preneoplastic cells show a moderate proliferative advantage over the phenotypically normal hepatocytes (Rabes & Szymkowiak, 1979). However, this is not reflected by the variation in the mitotic index (Barbason *et al.*, 1977). In spite of low variation in length of DNA synthesis (S phase) during the precancerous stage, the proliferative advantage can be expressed as an increasing LI after 7 injections of [$^{3}$H]dT at 6h intervals (Rabes & Szymkowiak, 1979). Moreover, the same authors show that, during the preneoplastic period, the proliferative fraction of phenotypically normal hepatocytes with a cell cycle shorter than 40 h increases up to ~ 75%.

Our present data corroborate these previous results. In similar experimental conditions, we have measured the proliferative fraction in normal and preneoplastic cells by using the same LI and estimated the cell content of the foci and their relative volumes.

In each of our 3 experimental groups (DEN for 2, 4 and 6 weeks) the LI in preneoplastic cells is higher than in the surrounding liver tissue. Moreover, the rate of proliferation in both types of cells depends on the duration of the DEN feeding. Indeed, preneoplastic areas of largest size ( > 200 cells/section) appear at the 3rd month after DEN for 6 weeks, the 9th month after DEN for 4 weeks and are not yet present 14 months after DEN for 2 weeks. The same type of correlation is found when estimating the relative volume of preneoplastic areas.

It must also be pointed out that in the 2 groups producing cancers the relatively low rate of proliferation during the preneoplastic stage is followed by an increasing proliferation in the preneoplastic areas after these have reached *critical size* (>200 cells/section, >40/1000 of relative volume). This situation corresponds to the moment when the neoplastic growth takes place, and when the homoeostatic control of cell proliferation is lost (Barbason *et al.*, 1977; Barbason & Betz, 1980). This notion of the critical size of islands, corresponding to the appearance of neoplastic nodules and to the disturbance of homoeostatic control, corroborate previous observations made during continuous DEN feeding (Rabes *et al.*, 1970, 1972, Rabes & Szymkowiak, 1979).

As previously shown (Heine & Morath, 1979; Hirota & Williams, 1979), the proliferative advantage of preneoplastic areas persists in our experimental conditions for many months after exposure to DEN. Therefore the phenomenon cannot be exclusively due to an increased resistance of preneoplastic cells to the toxicity of the carcinogen as previously proposed in other conditions (Farber *et al.*, 1976). The selective growth of preneoplastic lesions is rather due to the early carcinogen treatment. It has already been observed that neoplastic nodules and hepatocarcinomas arise a long time after cessation of a short carcinogen exposure sufficient to induce preneoplastic foci (Hirota & Williams, 1979).

It may be speculated how the early carcinogen treatment has a long-term influence on the disturbance of mitotic control, the proliferation of normal and precancerous lesions and the pathological evolution. It must be kept in mind that acute carcinogen treatment may induce mutations of different types which are distributed at random in the whole liver tissue. We have previously shown that, after a single nitrosamine injection, the lesions liable to develop into preneoplastic cells must be revealed at once by partial hepatectomy, their half-life being very short. By contrast, the lesions likely to lead to larger nuclear lesions, such as mitotic disturbances and the formation of micronuclei, remain latent and are still expressed when hepatectomy is much later (Barbason *et al.*, 1975). If these late-expressed nuclear lesions result in cell loss that disturbs any mitotic control mechanism, low proliferation may be maintained for a long time after removal of the carcinogen. On the other hand, it has been suggested (Heine & Morath, 1979) that a low level of proliferative stimuli potentiate the growth of preneoplastic cells with a shorter cell cycle. There is thus evidence that latent nuclear lesions induced by the first DEN administration are an important factor in success of the growth of preneoplastic lesions and their further malignant transformation.

According to these considerations, a 2-week DEN feeding, though sufficient to induce preneoplastic areas, would not be sufficient to reach a "growth pressure" permitting the evolution of preneoplastic lesions up to malignancy. On the other hand, when DEN feeding is prolonged, the growth pressure would be sufficient to express the malignancy, the sooner the longer the treatment.

### REFERENCES

Barbason, H. & Betz, E. H. (1980) Liver cell control after discontinuation of DENA feeding in hepatocarcinogenesis. *Eur. J. Cancer*, **17**, 149.

Barbason, H., Fridman-Manduzio, A. & Betz, E. H. (1975) Long term effect of a single dose of dimethylnitrosamine on the rat liver. *Z. Krebsforsch.*, **84**, 135.

Barbason, H., Fridman-Manduzio, A., Lelievre, P. & Betz, E. H. (1977) Variations of liver cell control during diethyl nitrosamine carcinogenesis. *Eur. J. Cancer*, **13**, 13.

Barbason, H., Smoliar, V., Fridman-Manduzio, A. & Betz, E. H. (1979*a*) Effects of the discontinuation of chronic feeding of diethylnitrosamine on the development of hepatomas in adult rats. *Br. J. Cancer*, **40**, 260.

Barbason, H., Smoliar, V. & van Cantfort, J. (1979*b*) Correlation of liver growth and function during liver regeneration and hepatocarcinogenesis. *Arch. Toxicol.* (Supp.), **2**, 157.

FARBER, E., PARKER, S. & GRUENSTEIN, M. (1976) The resistance of putative premalignant liver cell populations, hyperplastic nodules, to the acute cytotoxic effects of some hepatocarcinogens. *Cancer Res.*, **36,** 3879.

HEINE, W. D. & MORATH, R. (1979) Proliferation kinetics of preneoplastic foci in rat liver after partial hepatectomy. *Xth Meeting Eur. Study Group for Cell Proliferation.*

HIROTA, N. & WILLIAMS, G. M. (1979) Persistence of growth of rat liver neoplastic nodules following cessation of carcinogen exposure. *J. Natl Cancer Inst.*, **63,** 12.

RABES, H., HARTENSTEIN, R. & SCHOLZE, P. (1970) Specific stages of cellular response to homeostatic control during diethylnitrosamine-induced liver carcinogenesis. *Experientia*, **26,** 1356.

RABES, H. M., SCHOLZE, P. & YANTSCH, B. (1972) Growth kinetics of diethylnitrosamine induced enzyme deficient "preneoplastic" liver cell population *in vivo* and *in vitro*. *Cancer Res.*, **32,** 2577.

RABES, H. M. & SZYMKOWIAK, R. (1979) Cell kinetics of hepatocytes during the preneoplastic period of diethylnitrosamine induced liver carcinogenesis. *Cancer Res.*, **39,** 1298.

SQUIRE, R. A. & LEVITT, M. A. (1975) Report of a workshop on classification of specific hepatocellular lesions in rats. *Cancer Res.*, **35,** 3214.

WEIBEL, E. R. (1970) An automatic sampling stage microscope for stereology. *J. Microsc.* (*Oxf.*), **91,** 1.